# Spatial Metabolomic Profiling of Pinelliae Rhizoma from Different Leaf Types Using Matrix-Assisted Laser Desorption/Ionization Mass Spectrometry Imaging

**DOI:** 10.3390/molecules29174251

**Published:** 2024-09-07

**Authors:** Jiemin Wang, Xiaowei Han, Yuguang Zheng, Yunsheng Zhao, Wenshuai Wang, Donglai Ma, Huigai Sun

**Affiliations:** 1 College of Pharmacy, Hebei University of Chinese Medicine, Shijiazhuang 050200, China; wjm15030331834@163.com (J.W.); hanxiaowei2015@126.com (X.H.); zyg314@163.com (Y.Z.); zwhjzs@126.com (Y.Z.); wangwenshuai2003@126.com (W.W.); 2 Traditional Chinese Medicine Processing Technology Innovation Center of Hebei Province, Shijiazhuang 050200, China; 3 Key Laboratory for Quality Ensurance and Innovative TCMs of Dao-Di Herbs, Hebei Provincial Administration of Traditional Chinese Medicine, Shijiazhuang 050200, China

**Keywords:** Pinelliae Rhizoma, tissue distribution, secondary metabolite, MALDI-MSI, OPLS-DA, HPLC

## Abstract

Pinelliae Rhizoma (PR), a highly esteemed traditional Chinese medicinal herb, is widely applied in clinical settings due to its diverse pharmacological effects, including antitussive, expectorant, antiemetic, sedative-hypnotic, and antitumor activities. *Pinellia ternata* exhibits morphological variation in its leaves, with types resembling peach, bamboo, and willow leaves. However, the chemical composition differences among the corresponding rhizomes of these leaf phenotypes remain unelucidated. This pioneering research employed Matrix-Assisted Laser Desorption/Ionization Mass Spectrometry Imaging (MALDI-MSI) to conduct the in situ identification and spatial profiling of 35 PR metabolites in PR, comprising 12 alkaloids, 4 organic acids, 12 amino acids, 5 flavonoids, 1 sterol, and 1 anthraquinone. Our findings revealed distinct spatial distribution patterns of secondary metabolites within the rhizome tissues of varying leaf types. Orthogonal Partial Least Squares Discriminant Analysis (OPLS-DA) effectively differentiated between rhizomes associated with different leaf morphologies. Furthermore, this study identified five potential differential biomarkers—methylophiopogonanone B, inosine, cytidine, adenine, and leucine/isoleucine—that elucidate the biochemical distinctions among leaf types. The precise tissue-specific localization of these secondary metabolites offers compelling insights into the specialized accumulation of bioactive compounds in medicinal plants, thereby enhancing our comprehension of PR’s therapeutic potential.

## 1. Introduction

Pinelliae Rhizoma (PR), the dried rhizome of *Pinellia ternata* (Thunb.) Ten. ex Breitenb., has long been a staple in traditional medicine practices in China, Japan, and Korea since its first recorded use in the “Classic of Shennong Materia Medica” [1,2]. Its widespread application is attributed to its broad spectrum of therapeutic effects, including antidiarrheal, hypolipidemic, anti-tumor, antitussive, antiemetic, expectorant, and anti-gastric ulcer effects [3,4]. Phenotypic diversity within *P. ternata* populations is notable, persisting across consistent or varied environmental conditions, with the most significant variation observed in leaf morphology. This has led to the classification of three distinct leaf types: peach-leaf (PT), bamboo-leaf (BT), and willow-leaf (WT) [5,6].

To date, over 200 compounds have been discovered in PR, including alkaloids, volatile oils, amino acids, organic acids, and flavonoids [7,8,9,10,11]. However, the variations in chemical composition among PRs with different leaf morphologies have not been extensively investigated, which limits our comprehensive understanding of their medicinal properties. Therefore, it is crucial to elucidate the content and distribution of bioactive components within PRs from various leaf morphologies.

High-performance liquid chromatography (HPLC) and liquid chromatography-mass spectrometry (LC-MS) are standard methods for analyzing drug composition and for qualitative and quantitative assessments of drug extracts [12,13,14]. However, the microlocalization of components within the sample is often neglected during the sample pretreatment process, which can result in the loss of metabolite content in the extract and pose challenges for detection. Moreover, these techniques do not provide a visual depiction of the spatial distribution of metabolites within plant tissues. Therefore, the development of an in situ method to capture spatial distribution information on PR metabolites is imperative.

The mass spectrometry imaging (MSI) technology has enabled the visualization of multi-component analyses on tissue surfaces. The application of this technique in analyzing the secondary metabolites of medicinal plants has garnered significant interest in recent years [15,16,17]. The principal ionization sources for MSI are Matrix-Assisted Laser Desorption/Ionization (MALDI), Desorption Electrospray Ionization (DESI), and Secondary Ion Mass Spectroscopy (SIMS) [18]. Only MALDI and DESI are conducive to in situ analysis of small molecules. MALDI offers a spatial resolution of 1.4 μm and is the most widely used ion source in MSI [19]. In contrast, DESI provides several benefits, such as simplified sample preparation and the absence of a need for a matrix. However, its spatial resolution is limited to approximately 200 μm, which diminishes its applications and necessitates high spatial precision compared to other imaging techniques [20].

MALDI-MSI is a sophisticated technique for in situ molecular analysis that employs a soft ionization method. This method offers several advantages: (1) it requires minimal sample preparation without the need for extraction; (2) it provides high resolution, with spatial resolution up to 10 μm; and (3) it allows for in situ analysis without preliminary labeling [21]. This technique has been successfully used to characterize metabolites and secondary metabolites in various plants, such as *Ginkgo biloba* [22], *Panax ginseng* [23], and *Ligustri lucidi* [24]. Nonetheless, it is important to acknowledge the limitations of MALDI-MSI, such as potential matrix effects that can influence ionization efficiency and a limited dynamic range for quantitative analysis [17]. To circumvent these limitations, this study integrates MALDI-MSI and high-performance liquid chromatography (HPLC), thereby combining the high spatial resolution of MALDI-MSI with the quantitative precision of HPLC for a more comprehensive investigation.

The current study represents the first comprehensive imaging of 35 constituents, including alkaloids, organic acids, flavonoids, amino acids, sterols, and anthraquinones, in PRs from three distinct leaf types using MALDI-MSI. The contents of eight alkaloids were quantified using HPLC methods. Additionally, chemometric analysis was conducted to differentiate PRs from various leaf types and to identify discriminant compounds, aiming at ascertaining high-quality PR resources. This study supplies valuable references for the extraction, isolation, and identification of PR metabolites from different leaf types, as well as exploration of their spatial distribution.

## 2. Results

### 2.1. Comparative Analysis of Main Agronomic Traits and MALDI-TOF MS Investigation of PR

**Figure 1:**
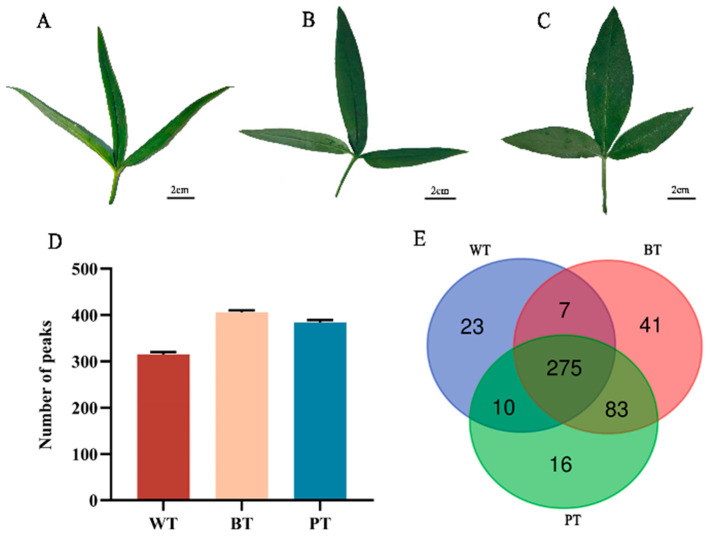
Macroscopic leaf characteristics and associated rhizome metabolite analysis in *P. ternata*: (**A**) WT leaves; (**B**) BT leaves; (**C**) PT leaves; (**D**) quantitative comparison of features found in the WT, BT, and PT rhizomes; (**E**) a Venn diagram representing the shared and unique feature counts among the rhizomes of the three leaf types.

This investigation conducts a comparative analysis of the principal agronomic traits of *P. ternata*, categorizing the plants into three distinct leaf types based on leaf morphology and the ratio of mid-leaf length to width: PT (4:1), BT (7:1), and WT (12:1). Figure 1A–C illustrate the representative leaf shapes for each type, and Table 1 details the comparative analysis of leaf area per plant and rhizome fresh weight. PT exhibited the largest average leaf area (16.379 cm^2^) and also had the highest average rhizome fresh weight (1.865 g per plant). In contrast, the WT demonstrated the lowest values for these parameters. PT displayed a significantly greater rhizome fresh weight per unit leaf area.

Using Matrix-Assisted Laser Desorption/Ionization Time-of-Flight Mass Spectrometry (MALDI-TOF MS), the rhizomes from each of the three leaf types of *P. ternata* were examined independently. The metabolic profiling uncovered a diverse spectrum of metabolites, identifying 315 features in WT rhizomes, 406 in BT rhizomes, and 384 in PT rhizomes. A notable overlap was observed, with 275 features being common across all types (Figure 1D,E). Detailed features can be found in the Supplementary Materials. Targeted analysis and accurate molecular weight measurements facilitated the identification of 35 specific metabolite components. These components belonged to various metabolite classes, including 12 alkaloids, 4 organic acids, 12 amino acids, 5 flavonoids, 1 sterol, and 1 anthraquinone (Table 2).

### 2.2. MALDI-MSI Analysis of Alkaloid Distribution in PRs

**Figure 2:**
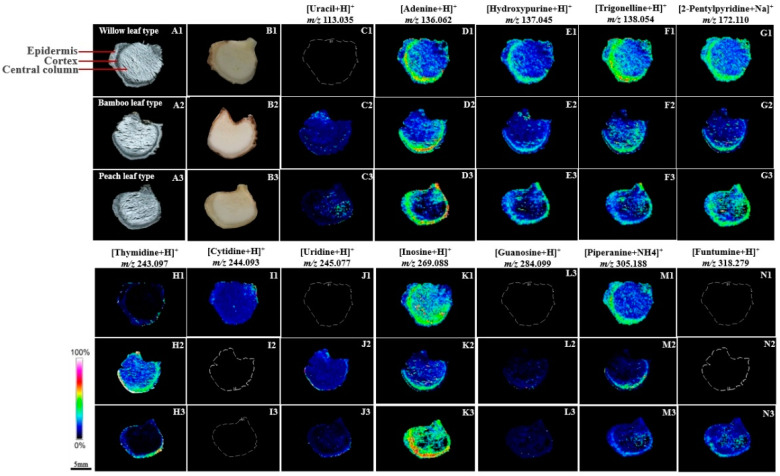
MALDI-MSI analysis of alkaloids in PRs: (**A1**–**A3**,**B1**–**B3**) sectional structure of PRs; (**C1**–**C3**)–(**N1**–**N3**) representative ion images of selected regions, with relative distribution depicted as a heat map ranging from blue (0%) to pink (100%).

Figure 2 presents a detailed visualization of the spatial distribution of twelve alkaloids across PRs, as determined by MALDI-MSI. The rhizome sections were categorized into three distinct anatomical regions for analysis: the central column, cortex, and epidermis (Figure 2(A1–A3,B1–B3)). Each region exhibits a distinctive alkaloid distribution pattern. These alkaloids were identified as molecular ions, including [M+H]^+^, [M+Na]^+^, and other specific adducts, based on their unique *m*/*z* values.

Thymidine (*m*/*z* 243.097, Figure 2(H1–H3)) was found to be broadly distributed in the BT rhizomes, with particularly high concentrations in the cortical region. In contrast, in the WT and PT rhizomes, thymidine was predominantly localized to the epidermis and cortex, with minimal presence in the central column. Adenine (*m*/*z* 136.062, Figure 2(D1–D3)), hydroxypurine (*m*/*z* 137.045, Figure 2(E1–E3)), trigonelline (*m*/*z* 138.054, Figure 2(F1–F3)), inosine (*m*/*z* 269.088, Figure 2(K1–K3)), 2-pentylpyridine (*m*/*z* 172.110, Figure 2(G1–G3)), and piperanine (*m*/*z* 305.229, Figure 2(M1–M3)) exhibited similar distribution patterns across the WT, BT, and PT rhizomes, being nearly ubiquitous throughout the tissue sections, with a pronounced concentration in the epidermis and cortex.

Uracil (*m*/*z* 113.031, Figure 2(C1–C3)), uridine (*m*/*z* 245.077, Figure 2(J1–J3)), and guanosine (*m*/*z* 284.099, Figure 2(L1–L3)) were detected in the BT and PT rhizomes but were absent in the WT rhizomes. Cytidine (*m*/*z* 244.093, Figure 2(I1–I3)) was exclusively detected in the WT rhizome sections, with no evidence of its presence in the BT and PT rhizomes. Additionally, funtumine (*m*/*z* 318.249, Figure 2(N1–N3)) was specifically localized to the PT rhizomes and was not detected in the WT and BT rhizomes. Uracil and uridine were predominantly distributed in the epidermis and central column of the PT rhizomes while exhibiting a more uniform distribution across the BT section. In contrast, guanosine was sparsely distributed throughout the PT rhizomes and was also found in low abundance in the lower part of the BT sections.

### 2.3. Distribution of Organic Acids in PRs

**Figure 3:**
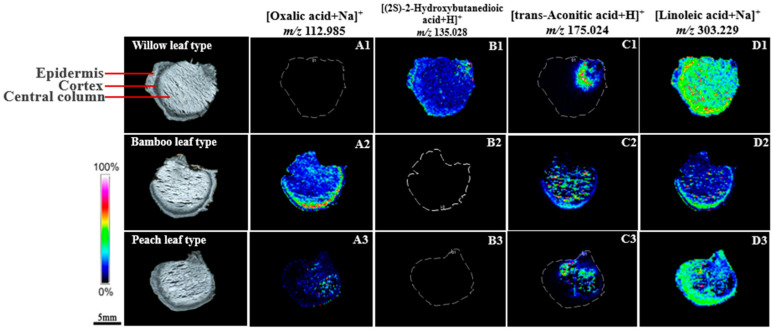
MSI of organic acids in PR reveals differential distribution within rhizome sections, categorized into epidermis, cortex, and central column. Heat map with color gradient from blue (0%) to pink (100%) visualizes relative compound abundance. (**A1**–**A3**)–(**D1**–**D3**): A representative ion image of the selected region.

Examination of Figure 3 illustrates distinct distribution patterns of organic acids within various types of PRs. (2*S*)-2-hydroxybutanedioic acid, with a mass-to-charge ratio (*m*/*z*) of 135.028 (Figure 3(B1–B3)), exhibited a uniform distribution across the WT rhizome. In the BT rhizome, oxalic acid (*m*/*z* 112.985, Figure 3(A2)), trans-aconitic acid (*m*/*z* 175.024, Figure 3(C2)), and linoleic acid (*m*/*z* 303.229, Figure 3(D2)) were predominantly localized to the epidermal and cortical tissues, suggesting a consistent distribution profile. Comparative analysis revealed that linoleic acid (*m*/*z* 303.229, Figure 3(D1–D3)) was also relatively abundant in the epidermal and cortical regions of both WT and PT rhizomes. In the PT rhizome, oxalic acid (*m*/*z* 112.985, Figure 3(A3)) and trans-aconitic acid (*m*/*z* 175.024, Figure 3(C3)) displayed a distribution similar to that in the WT rhizome, predominantly located in the upper right section of the tissue, adjacent to the rhizome’s connection to the aerial parts.

### 2.4. MALDI-MSI Imaging of Amino Acids in PRs

**Figure 4:**
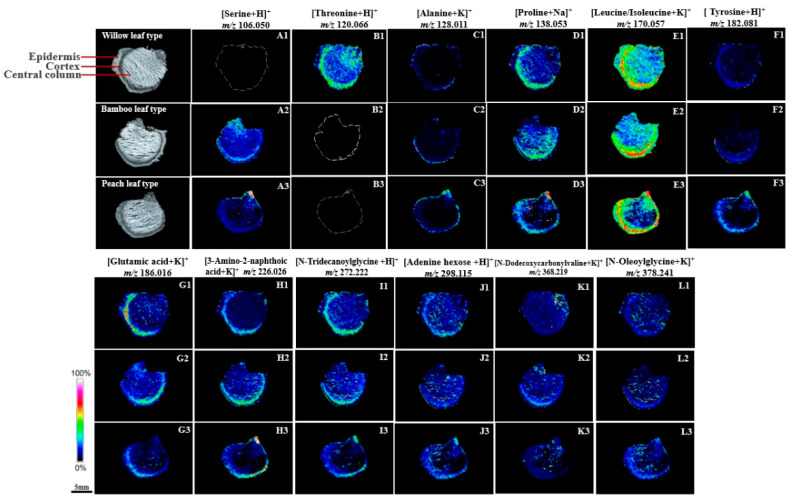
MSI delineates the tissue-specific distribution of amino acids in PR, with the legend indicating ion intensity and the color gradient reflecting expression levels. Scale bar: 5 mm. (**A1**–**A3**)–(**L1**–**L3**): A representative ion image of the selected region.

The distribution patterns of amino acids such as proline (*m*/*z* 138.053, Figure 4(D1–D3)), leucine/isoleucine (*m*/*z* 170.057, Figure 4(E1–E3)), glutamic acid (*m*/*z* 186.016, Figure 4G1–G3), 3-amino-2-naphthoic acid (*m*/*z* 226.026, Figure 4(H1–H3)), *N*-tridecanoylglycine (*m*/*z* 272.222, Figure 4(I1–I3)), adenine hexose (*m*/*z* 298.115, Figure 4(J1–J3)), *N*-dodecoxycarbonylvaline (*m*/*z* 368.219, Figure 4(K1–K3)), and *N*-oleoylglycine (*m*/*z* 378.241, Figure 4(L1–L3)) were similar across the WT, BT, and PT rhizomes, with primary localization in the epidermis and cortex. These eight amino acids were distributed throughout the sections in the WT and BT, whereas their presence in the central column of PT was minimal. Alanine (*m*/*z* 128.011, Figure 4(C1–C3)) and tyrosine (*m*/*z* 182.081, Figure 4(F1–F3)) were detected only in the epidermis and cortex across all three rhizome types, with no detection in the central column. Serine (*m*/*z* 106.050, Figure 4(A1–A3)) was present in the BT and PT but absent in the WT. Threonine (*m*/*z* 120.066, Figure 4(B1–B3)) was detected in the WT but not in the BT and PT. The epidermis and cortex exhibited high concentrations of all these amino acids. In particular, serine in the PT (Figure 4(A3)) showed elevated distribution in the region connecting the rhizome to the above-ground part.

### 2.5. MALDI-MSI Imaging of Flavonoids and Other Metabolites in PRs

**Figure 5:**
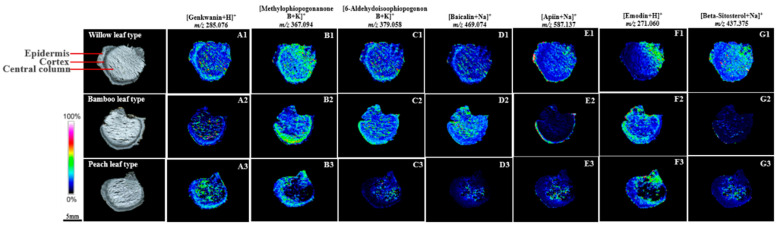
MSI unveils the distribution of flavonoids and other metabolites in PR, with the legend representing ion relative intensity and the color gradient indicating expression levels. Scale bar: 5 mm. (**A1**–**A3**)–(**G1**–**G3**): A representative ion image of the selected region.

In the WT rhizome, flavonoids such as genkwanin (*m*/*z* 285.076, Figure 5(A1)), methylophiopogonanone B (*m*/*z* 367.094, Figure 5(B1)), 6-aldehydoisoophiopogonon B (*m*/*z* 379.058, Figure 5(C1)), and Beta-sitosterol (*m*/*z* 437.375, Figure 5(G1)), baicalin (*m*/*z* 469.074, Figure 5(D1)), and apiin (*m*/*z* 587.137, Figure 5(E1)), were widely distributed throughout the rhizome section, with a notable concentration in the cortex. Emodin (*m*/*z* 271.060, Figure 5(F1)), however, displays a distinct distribution pattern, being less abundant in superficial tissues and more concentrated near the rhizome’s aerial attachment. Similar distribution patterns are observed in the BT rhizome, with emodin (Figure 5(F2)) and genkwanin (Figure 5(A2)) being more prevalent in the epidermis and cortex, while Beta-sitosterol (Figure 5(G2)) showed a more centralized distribution. In the PT rhizome, a shift was observed, with genkwanin (Figure 5(A3)) and 6-aldehydoisoophiopogonon B (Figure 5(C3)) being more pronounced in the central column, whereas emodin (Figure 5(F3)) and methylophiopogonanone B (Figure 5(B3)) maintained their preference for the epidermal and cortical regions.

### 2.6. Chemical Composition Analysis of PRs with Various Leaf Types

**Figure 6:**
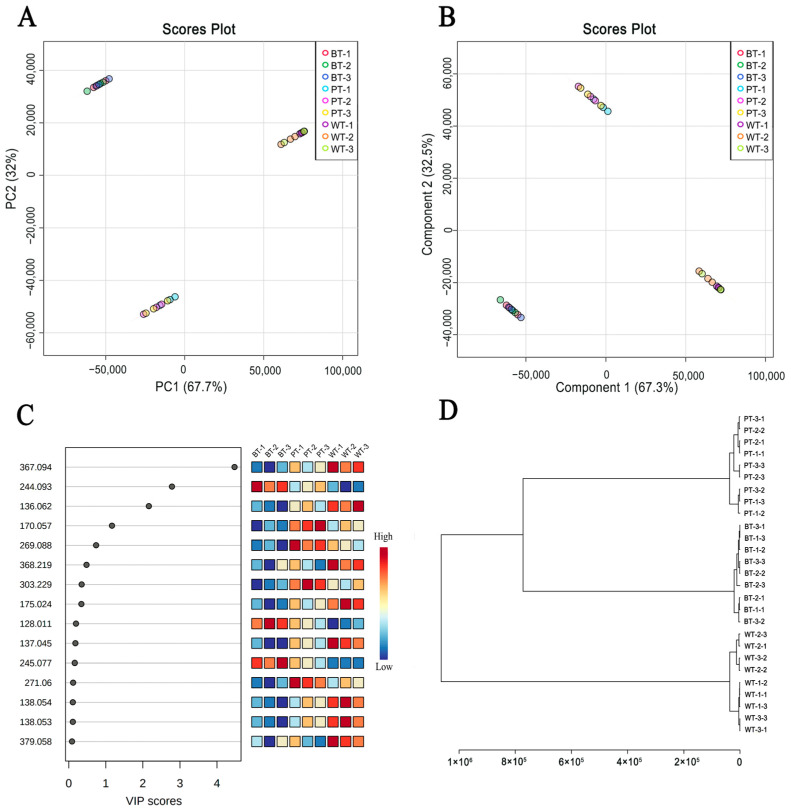
Multivariate analysis revealing chemical patterns in PRs of different leaf morphotypes. (**A**) PCA score plot. (**B**) OPLS-DA model score plot. (**C**) Sample VIP value plot. The gradient scale transitions from dark blue to dark red, corresponding to an increase from lower to higher levels of expression. (**D**) Sample hierarchical clustering dendrogram. The ruler at the bottom of the dendrogram shows the distance between samples.

Principal Component Analysis (PCA) revealed that the first principal component contributed 67.7% of the variance, while the second principal component accounted for 32.0%, with a combined cumulative contribution rate of 99.7%. This high cumulative contribution rate suggests that the PCA model developed has a strong discriminative ability, effectively capturing the primary chemical characteristics of the PR samples across different leaf types. Based on these two principal components, a coordinate system was constructed, and a PCA score plot was generated for 27 batches of PR samples from the three distinct leaf types (Figure 6A). The score plot shows that samples of each leaf type cluster in specific areas, indicating significant differences among the groups.

To further investigate the chemical composition differences and dynamics of these variations among PRs of different leaf types, a supervised Orthogonal Partial Least Squares Discriminant Analysis (OPLS-DA) was conducted following the initial unsupervised PCA. The OPLS-DA score plot (Figure 6B) is consistent with the PCA results, with the 27 batches of PR samples clearly separated into three categories, matching their respective leaf morphological characteristics. Cluster analysis (Figure 6D) similarly partitioned the samples into three categories, corresponding to the leaf morphological characteristics, with BT and PT showing a closer phylogenetic relationship.

Through comprehensive differential analysis, we obtained Variable Importance in Projection (VIP) sores for the variable weights. Based on the VIP values of the compounds within the model, those with significant differences were identified, and compounds with VIP values greater than 1.0 were selected as potential chemical markers. Among the 35 components identified, methylophiopogonanone B, cytidine, adenine, and leucine/isoleucine had VIP values greater than 1 (Figure 6C), indicating that these compounds may serve as key indicators of the chemical distinctions among PR samples of different leaf types.

### 2.7. Heat Map Analysis of PR Compounds

**Figure 7:**
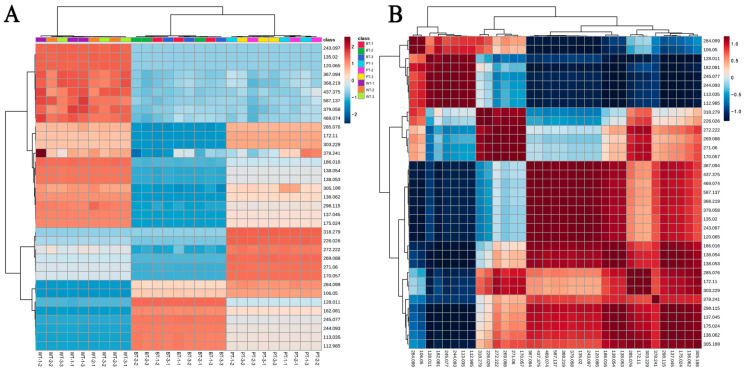
Heat map analyses portray compound composition and correlation intricacies in PRs. (**A**) Cluster heat map analysis illustrates variations in compound profiles across WT, BT, and PT rhizomes, with the horizontal axis representing samples and the vertical axis listing *m*/*z* values. Expression levels are signified by a color gradient from dark blue (low) to dark red (high). (**B**) Correlation heat map analysis delineates compound interrelationships, with the same gradient indicating the degree of correlation.

Heat map clustering showed a closer phylogenetic relationship between the PT and BT samples. The MSI detection revealed variability in the responsiveness of active constituents across the WT, PT, and BT samples. The WT samples contained eighteen compounds, characterized by their *m*/*z* ratios, which demonstrated an enhanced response to MSI. By comparison, PT and BT samples had eleven and six compounds, respectively, which exhibited a strong response. Seven compounds in BT samples, which included flavonoids, sterols, and anthraquinones, consistently exhibited diminished MSI signals (Figure 7A). Correlation analysis indicated that 22 compounds had significant intercorrelations, suggesting complex metabolic interactions. In particular, the following pairs showed strong associations: serine and guanosine, *N*-tridecanoylglycine and genkwanin, and emodin and 2-pentylpyridine (Figure 7B).

### 2.8. Quantitative Analysis of PRs

**Figure 8:**
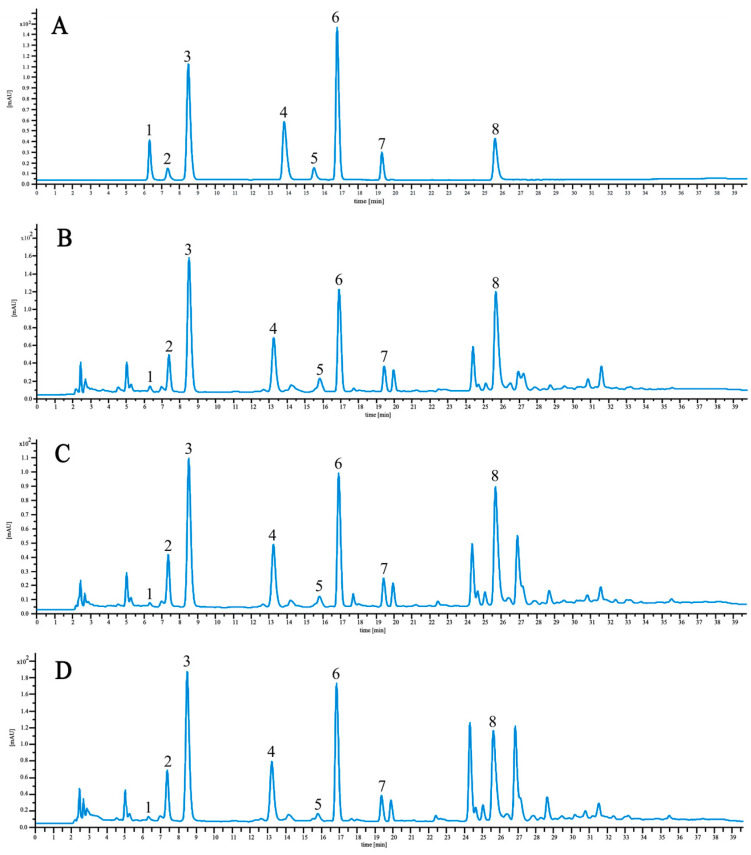
HPLC chromatograms of a reference compound (**A**) and PRs of WT (**B**), BT (**C**), and PT (**D**). Peaks correspond to 1. uracil, 2. cytidine, 3. uridine, 4. hydroxypurine, 5. inosine, 6. guanosine, 7. thymidine, and 8. adenine.

We conducted a 40 min analysis on rhizome samples of *P. ternata*, which were categorized by leaf types. Figure 8 displays the distribution maps of alkaloidal constituents in the WT, BT, and PT rhizomes, identifying eight alkaloids with considerable variations in content across the leaf types (Table 3). Adenine was the most abundant compound, with concentrations measured at 824.087, 622.910, and 804.092 μg·g^−1^ in the WT, BT, and PT rhizomes, respectively. In contrast, uracil was the least abundant, with concentrations of 11.579, 4.091, and 10.284 μg·g^−1^ in the respective rhizomes.

The BT rhizome exhibited a general decrease in alkaloidal content, with individual quantities ranging from 4.091 to 622.910 μg·g^−1^. Conversely, the PT rhizome displayed elevated levels of cytidine, uridine, hydroxypurine, guanosine, and thymidine, quantified at 247.228, 498.725, 171.601, 481.643, and 183.231 μg·g^−1^, respectively. Additionally, the WT rhizome contained higher levels of uracil, inosine, and adenine, with quantities significantly exceeding those in the PT and BT rhizomes (Table 4).

### 2.9. OPLS-DA Analysis of Eight Alkaloidal Components in PRs

**Figure 9:**
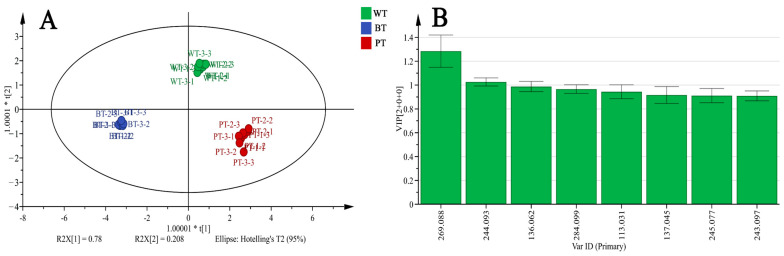
OPLS-DA score plot (**A**) and VIP score plot (**B**) of eight alkaloid components in PR samples across distinct leaf morphotypes. The analysis was conducted using triplicate biological samples with nine technical replicates each.

We conducted a statistical analysis using HPLC data to characterize the chemical profiles of PR samples across various leaf morphotypes. The OPLS-DA model effectively differentiated the three leaf types (Figure 9A). To assess the impact of individual alkaloidal components on the model’s discriminative ability, we used the VIP score, using a cutoff of >1 to identify key differentiating compounds. Inosine (*m*/*z* 269.088) and cytidine (*m*/*z* 244.093) yielded the highest VIP scores, 1.29 and 1.12, respectively (Figure 9B), suggesting their potential as the most discriminative components among the three PR samples.

## 3. Discussion

Factors such as sample preparation, substrate selection, and ion source choice can influence the technique’s outcomes. Early studies have demonstrated that 2-mercaptobenzothiazole (2-MBT) provides sensitivity and resolution comparable to other matrices, with an added advantage of higher tolerance for contaminants, such as ionic detergents [25]. It was found that 2-MBT exhibited the least background peak interference and the best detection performance for metabolic molecule ion signals during the evaluation of the detection performance of α-cyano-4-hydroxycinnamic acid (DHB), α-cyano-4-hydroxy-3-methylcinnamic acid (CHCA), and 2-MBT as MALDI matrices on rock orchid leaf tissue [26]. Schwartz et al. [27] recommended the immediate freezing of samples in liquid nitrogen for 30 to 60 s post-sampling to preserve tissue shape and chemical composition. They also reported samples stored at −80 °C showed no significant degradation up to one year. Therefore, the PRs in this study were frozen in liquid nitrogen, sectioned at a precise temperature, and uniformly coated with the 2-MBT matrix before undergoing detection and analysis by MALDI-MSI.

Rhizome yield is a critical factor in increasing the production and income of *P. ternata*, making it a significant cultivation indicator [28]. We found that the rhizome fresh weight of the PT exceeded that of the BT and the WT. Traits such as leaf mass per unit area, nitrogen content per unit leaf area, maximum carboxylation capacity, and the ratio of leaf internal to ambient CO_2_ partial pressure are essential for understanding leaf photosynthetic function [29]. PT demonstrated a higher rhizome fresh weight per unit of leaf area, suggesting a more efficient accumulation of photosynthetically active products, likely due to its larger leaf size. Gray correlation analysis showed that PR alkaloids are the most potent component regarding their cough-suppressant and expectorant effects, serving as a quality control indicator [30]. These alkaloids may exert antiemetic effects by blocking 5-HT3 and NK1 receptors [31]. The quantification of eight representative alkaloid contents by HPLC showed that PT and WT contained higher concentrations of all eight alkaloids compared to BT. Although the WT had higher concentrations of the eight alkaloids, its rhizome fresh weight was significantly lower than that of PT. Hence, it is hypothesized that PT is the optimal choice for cultivation, within the controllable range of alkaloid toxicity.

Numerous studies have identified a total of 212 distinct compounds extracted and characterized from PR, including alkaloids, volatile oils, amino acids, organic acids, flavonoids, cerebrosides, and phenylpropanoids. Among these compounds, alkaloids, flavonoids, and organic acids were the most abundant and displayed significant biological activities. They have emerged as prime candidates for further research [32,33,34,35]. Lange et al. [36] reported that sesquiterpene pyridine alkaloids were primarily located in the cortex of the transverse root slice of *Tripterygium wilfordii*. Similarly, He et al. [37] found that alkaloids such as acetyltropine and protopine were prevalent in the inner seed coat of *Taxus chinensis*. These findings align with the results of the present study, suggesting that alkaloids are more abundant in the epidermis and cortex, leading to the hypothesis that these tissues are rich in nitrogen-containing compounds. Future research could involve targeted extraction of compounds from specific regions of PR to validate findings, potentially leading to cost savings and enhanced resource utilization. Furthermore, it was observed that individual compounds, such as emodin (*m*/*z* 271.060), methylophiopogonanone B (*m*/*z* 367.094), and trans-aconitic acid (*m*/*z* 175.024) in the WT and PT, were more distinctly distributed at the junction of rhizome and the above-ground part of the plant. This suggests that the above-ground part may be a new source of medicinal compounds.

OPLS-DA is a multivariate analysis method that effectively captures differences between sample groups, predicts the grouping of samples, and identifies the most important classification variables [38,39]. We used OPLS-DA to analyze both MALDI-MSI and HPLC data, and the results indicated clear differences among PRs of different leaf types in both experimental datasets. The VIP values facilitated the screening of different metabolites and the identification of compounds with significant differences across the three sample groups: methylophiopogonanone B, inosine, cytidine, adenine, and leucine. These compounds effectively distinguished the PRs. Statistical analysis results indicated that certain compounds can serve as differential markers for different root and stem types. Among these compounds, the majority are the most abundant primary metabolites, namely nucleosides and amino acids. The speculated reason for this may be that nucleosides and amino acids are the most fundamental building blocks within living organisms, participating in the synthesis of DNA/RNA and proteins, respectively. They occupy a central position in cellular metabolism; hence, variations in their concentrations may have indicative significance for the physiological status of the plant and the differentiation of tissue types [40].

Methylophiopogonanone B (MO-B), a homoisoflavone monomer extracted from *Ophiopogon japonicus*, has been demonstrated to possess antioxidant and antitumor properties. MO-B has been found to protect human umbilical vein endothelial cells from H_2_O_2_-induced injury, and its protective effect may be mediated through the NADPH pathway. Furthermore, MO-B has been shown to attenuate H_2_O_2_-induced apoptosis by regulating the expression of apoptosis-related genes and proteins, such as Bax/Bcl-2 and caspase-3 [41]. Inosine, a nucleotide, has been identified as an alternative metabolic substrate for T cells, supporting CD8+ T cell proliferation in glucose-deficient conditions. The metabolites of inosine, including ATP and ribose phosphate, can provide energy and biosynthetic precursors for T cells. Additionally, inosine has been reported to enhance the anti-tumor effects of checkpoint blockade therapy or over-the-counter T-cell therapy by promoting T-cell-mediated tumor-killing activity [42]. The presence and concentration of these compounds (i.e., MO-B, inosine, cytidine, adenine, and leucine) showed significant differences among the WT, PT, and BT samples. These differences may reflect the unique biological properties and potential therapeutic applications of the samples. For example, the high concentration of MO-B in the WT may be associated with its strong antioxidant capacity, while high concentrations of inosine may be associated with an enhanced immune response. The findings of this study provide a foundation for further exploration of the role of these compounds in various biological processes.

The expression levels of the eight alkaloids in PRs from different leaf types were not identical to those determined by HPLC analysis. This discrepancy may arise from the fact that MALDI-MSI analyzes the local distribution of compounds in sections of PR with a thickness of 20 µm [43,44,45], whereas HPLC quantifies the compounds in the entire rhizome [46,47,48]. To understand the spatial distribution of alkaloids in the entire tuber, experiments conducted at a later stage could involve 2D mass spectrometry imaging and the stacked reconstruction of a series of tissue sections. This approach can construct a complete 3D mass spectrometry image of the tissue, thus elucidating the distribution of biomolecules within the complex biological structure. However, when multiple tissue sections need to be analyzed, conventional 3D MSI can be time-consuming. In this context, innovative approaches, such as the DeepS workflow, have shown significant progress. By employing a 3D sparse sampling neural network, DeepS is capable of achieving faster imaging speeds without compromising on imaging quality, as demonstrated by its application in the mouse brain and kidney datasets [49]. The complementary use of MALDI-MSI and HPLC enables a more detailed study of alkaloid profiles in the PR, and this integrated approach allows for the comprehensive characterization of PR alkaloids, reflecting the unique contributions of each analytical technique.

## 4. Materials and Methods

### 4.1. Reagents and Materials

This study adhered to institutional, national, and international guidelines and regulations for the cultivation of *P. ternata*. The three variants of *P. ternata*, distinguished by their leaf morphologies—peach-leaf, bamboo-leaf, and willow-leaf—were authenticated by Prof. Yuguang Zheng at the Shijiazhuang Traditional Chinese Medicine Processing Technology Innovation Center, Hebei Province, China. Voucher specimens of the plant and medicinal materials have been deposited at the herbarium of Hebei University of Chinese Medicine (Voucher Number: 23061801027LY, 23061801028LY, and 23061801029LY).

HPLC-grade solvents, including methanol and acetonitrile, were sourced from Merck & Co., Inc. (Darmstadt, Germany). The Optimum Cutting Temperature (OCT) compound and trifluoroacetic acid (TFA) were obtained from Sigma-Aldrich (St. Louis, MO, USA), and purified water was generated using a Milli-Q filtration system (Bedford, MA, USA). Standard compounds were procured from Beijing Solarbio Science & Technology Co., Ltd., Beijing, China. Detailed information on batch numbers and preparation concentrations of these standards is presented in the Supplementary Materials.

### 4.2. Determination of Rhizome Rresh Weight and Leaf Area Measurement

Prior to weight assessment, *P. ternata* plants with distinct leaf types were cleaned and dried. Leaf areas were digitally measured using Photoshop 2021 (https://www.adobe.com/products/photoshop.html (accessed on 3 April 2024)) with a 1 cm^2^ grid as reference. Six plants per leaf type with uniform growth were selected for triplicate measurements of leaf area and rhizome weight. The leaf area was calculated using the following formula: leaf area = (reference area × leaf pixel count)/reference pixel count. Data are presented as mean ± SD and were analyzed using SPSS 26.0 (https://www.ibm.com/support/pages/downloading-ibm-spss-statistics-26 (accessed on 8 April 2024)), with statistical differences assessed by one-way analysis of variance and Tukey’s test (*p* < 0.05).

### 4.3. Tissue Sectioning

Using a cryostat (Model CM1860, Leica Microsystems Inc., Nussloch, Germany), PR tissue samples were sectioned into 20 μm thick slices and transferred onto indium tin oxide (ITO)-coated conductive glass slides (Bruker Daltonics, Karlsruhe, Germany) for imaging and MALDI-MSI preparation. Optical imaging of these sections was conducted with a UMAX PowerLook III scanner (Umax Technologies, Fremont, CA, USA).

### 4.4. Matrix Application

A solution of 2-MBT was prepared at a concentration of 10 mg/mL using a solvent mixture composed of methanol and water in an 80:20 (*v*/*v*) ratio, supplemented with 0.2% TFA. The matrix application to the tissue sections of PR was carried out using an ImagePrep automated matrix spotter (Bruker Daltonics, Bremen, Germany). To ensure a uniformly thin substrate layer, the matrix solution was applied in a continuous spray for 5 s, followed by a 60 s drying period. This application and drying cycle were repeated for a total of 5 cycles. After the initial cycles and subsequent air drying in a fume hood, the matrix solution was evenly sprayed onto the tissue sections for an additional 40 cycles. The tissue sections, once coated, were then prepared for MALDI-MSI analysis.

### 4.5. MALDI-MSI Analysis

The profiling and imaging studies were conducted on an Autoflex Speed MALDI TOF/TOF mass spectrometer (Bruker Daltonics, Bremen, Germany), which was coupled with a high-repetition-rate solid-state Smartbeam Nd:YAG UV laser (355 nm) by Azura Laser AG. The profiling spectra covered a mass range of 100 to 2000 *m*/*z*, obtained from 40 scans at 500 laser shots each. Imaging of PR tissue sections with different leaf types was performed using a 200 μm laser raster step-size, with each pixel integrating 500 laser shots. FlexImaging 4.1 software (Bruker Daltonics, Bremen, Germany) was applied for precise UV laser targeting via “teaching points.”

Mass calibration employed a standard mixture for external calibration, comprising peptides with defined *m*/*z* values: bradykinin 1–7 ([M+H]^+^, *m*/*z* 757.40), angiotensin II ([M+H]^+^, *m*/*z* 1046.54), angiotensin I ([M+H]^+^, *m*/*z* 1296.69), substance P ([M+H]^+^, *m*/*z* 1347.74), and bombesin ([M+H]^+^, *m*/*z* 1619.82). Internal calibration referenced the matrix ion 2-MBT ([M+H]^+^, *m*/*z* 167.99) and a peptide standard ([M+H]^+^, *m*/*z* 1349.69), with the intensity of the peptide standard normalizing the spectral data. Calibration was conducted in cubic-enhanced mode to ensure the accuracy of mass measurements.

### 4.6. Data Analysis

MSI data were interpreted using Bruker FlexAnalyst 3.4 software (https://softwaretopic.informer.com/ (accessed on 22 April 2024)), with metabolite identification confirmed against the METLIN (https://metlin.scripps.edu/) and HMDB (https://hmdb.ca (accessed on 22 April 2024)) databases. The metabolite list generated by mass matching was further confirmed by comparison with purchased standards, the databases, or previous literature. A mass window is 0.3% and a signal-to-noise ratio is 3. Data visualization and statistical analysis were performed using Bruker FlexImage 4.1 and SIMCA 14.1 software (https://www.umetrics.com/ (accessed on 22 April 2024)), respectively. Heat maps were generated with TBtools (v 1.108) to depict compound abundance.

### 4.7. Extraction and Quantification of Eight Alkaloids by HPLC

Eight alkaloids were extracted from PRs using HPLC, and their content was quantified. The detailed extraction procedures and chromatographic conditions are provided in the Supporting Information.

## 5. Conclusions

This study pioneers the in situ examination of PR metabolite profiles from different leaf types using MALDI-MSI, yielding the spatial profiling of 35 compounds, including alkaloids, organic acids, amino acids, flavonoids, and other metabolites. Furthermore, eight alkaloids were quantified by HPLC, and OPLS-DA analysis identified five compounds that served as discriminatory markers, enabling rapid classification of the three PR leaf types.

## Figures and Tables

**Table 1:** Main agronomic traits of three distinct leaf-type *P. ternata*.

Leaf Shape	Leaf Area (cm^2^)	Fresh Weight of Rhizome (g)	Fresh Weight/Leaf Area (g/cm^2^)
PT	16.379 ^a^ ± 0.154	1.865 ^a^ ± 0.021	0.114 ^a^ ± 0.000
BT	13.427 ^b^ ± 0.135	1.383 ^b^± 0.019	0.103 ^b^ ± 0.000
WT	10.204 ^c^ ± 0.104	1.033 ^c^ ± 0.019	0.101 ^c^ ± 0.001

Notes: The table shows mean values and standard deviations. Mean values designated by different letters and placed in the same row differ and are statistically different at *p* < 0.05.

**Table 2:** Compounds with preliminary annotation by targeted MALDI-TOF MS analysis.

Classes	Formula	Adduct	Calculated Value (*m*/*z*)	Measured Value (*m*/*z*)	Error (ppm)	Preliminary Annotation
Alkaloids	C_4_H_4_N_2_O_2_	[M+H]^+^	113.0346	113.035	4	Uracil
	C_5_H_5_N_5_	[M+H]^+^	136.0618	136.062	1	Adenine
	C_5_H_4_N_4_O	[M+H]^+^	137.0458	137.045	6	Hydroxypurine
	C_7_H_7_NO_2_	[M+H]^+^	138.0550	138.054	7	Trigonelline
	C_10_H_15_N	[M+Na]^+^	172.1097	172.110	2	2-Pentylpyridine
	C_10_H_14_N_2_O_5_	[M+H]^+^	243.0975	243.097	2	Thymidine
	C_9_H_13_N_3_O_5_	[M+H]^+^	244.0928	244.093	1	Cytidine
	C_9_H_12_N_2_O_6_	[M+H]^+^	245.0768	245.077	1	Uridine
	C_10_H_12_N_4_O_5_	[M+H]^+^	269.0880	269.088	0	Inosine
	C_10_H_13_N_5_O_5_	[M+H]^+^	284.0989	284.099	0	Guanosine
	C_17_H_21_NO_3_	[M+NH_4_]^+^	305.1860	305.188	7	Piperanine
	C_21_H_35_NO	[M+H]^+^	318.2791	318.279	0	Funtumine
Organic acids	C_2_H_2_O_4_	[M+Na]^+^	112.9845	112.985	4	Oxalic acid
	C_4_H_6_O_5_	[M+H]^+^	135.0288	135.028	6	(2*S*)-2-Hydroxybutanedioic acid
	C_6_H_6_O_6_	[M+H]^+^	175.0237	175.024	2	trans-Aconitic acid
	C_18_H_32_O_2_	[M+Na]^+^	303.2295	303.229	2	Linoleic acid
Amino acids	C_3_H_7_NO_3_	[M+H]^+^	106.0499	106.050	1	Serine
	C_4_H_9_NO_3_	[M+H]^+^	120.0655	120.066	4	Threonine
	C_3_H_7_NO_2_	[M+K]^+^	128.0108	128.011	2	Alanine
	C_5_H_9_NO_2_	[M+Na]^+^	138.0525	138.053	4	Proline
	C_6_H_13_NO_2_	[M+K]^+^	170.0578	170.057	5	Leucine/Isoleucine
	C_9_H_11_NO_3_	[M+H]^+^	182.0812	182.081	1	Tyrosine
	C_5_H_9_NO_4_	[M+K]^+^	186.0163	186.016	2	Glutamic acid
	C_11_H_9_NO_2_	[M+K]^+^	226.0265	226.026	2	3-Amino-2-naphthoic acid
	C_15_H_29_NO_3_	[M+H]^+^	272.2220	272.222	0	*N*-Tridecanoylglycine
	C_11_H_15_N_5_O_5_	[M+H]^+^	298.1146	298.115	1	Adenine hexose
	C_18_H_35_NO_4_	[M+K]^+^	368.2198	368.219	2	*N*-Dodecoxycarbonylvaline
	C_20_H_37_NO_3_	[M+K]^+^	378.2405	378.241	1	*N*-Oleoylglycine
Flavonoids and others	C_16_H_12_O_5_	[M+H]^+^	285.0757	285.076	1	Genkwanin
	C_19_H_20_O_5_	[M+K]^+^	367.0942	367.094	1	Methylophiopogonanone B
	C_19_H_16_O_6_	[M+K]^+^	379.0578	379.058	1	6-Aldehydoisoophiopogonon B
	C_21_H_18_O_11_	[M+Na]^+^	469.0741	469.074	0	Baicalin
	C_26_H_28_O_14_	[M+Na]^+^	587.1371	587.137	0	Apiin
	C_15_H_10_O_5_	[M+H]^+^	271.0601	271.060	0	Emodin
	C_29_H_50_O	[M+Na]^+^	437.3754	437.375	1	Beta-Sitosterol

**Table 3:** Regression data for the quantitative analysis of eight alkaloidal components.

Analyte	Linear Range/(mg·mL^−1^)	Regression Equation	*R* ^2^
Uracil	3.2750~9.8250	Y = 53.432 X + 1.970	0.9993
Cytidine	2.5000~7.5000	Y = 27.911 X + 1.502	0.9994
Uridine	14.8750~44.6250	Y = 52.107 X + 9.944	1.0000
Hydroxypurine	6.6875~20.0625	Y = 66.163 X + 10.118	1.0000
Inosine	2.6000~7.8000	Y = 29.816 X + 3.388	0.9996
Guanosine	19.5000~58.5000	Y = 43.452 X + 12.234	1.0000
Thymidine	7.0625~21.1875	Y = 19.523 X + 2.658	0.9999
Adenine	13.8750~41.6250	Y = 22.005 X − 3.414	0.9994

**Table 4:** Sample determination results of alkaloidal components (μg·g^−1^).

Sample	Uracil	Cytidine	Uridine	Hydroxypurine	Inosine	Guanosine	Thymidine	Adenine
WT	11.579 ^a^ ± 0.121	163.748 ^b^ ± 0.243	411.560 ^b^ ± 0.324	140.920 ^b^ ± 0.135	94.946 ^a^ ± 0.095	340.844 ^b^ ± 0.353	168.039 ^b^ ± 0.131	824.087 ^a^ ± 0.532
BT	4.091 ^c^ ± 0.074	147.185 ^c^ ± 0.213	284.880 ^c^ ± 0.233	102.966 ^c^ ± 0.103	41.596 ^c^ ± 0.043	271.715 ^c^ ± 0.276	114.799 ^c^ ± 0.094	622.910 ^c^ ± 0.479
PT	10.284 ^b^ ± 0.132	247.228 ^a^ ± 0.295	498.725 ^a^ ± 0.325	171.601 ^a^ ± 0.113	45.621 ^b^ ± 0.046	481.643 ^a^ ± 0.401	183.231 ^a^ ± 0.152	804.092 ^b^ ± 0.586

Notes: The table shows mean values and standard deviations. Mean values designated by different letters and placed in the same row differ and are statistically significant at *p* < 0.05.

## Data Availability

The data presented in this study are available in the article and Supplementary Materials.

## Supplementary Materials

The following supporting information can be downloaded at https://www.mdpi.com/article/10.3390/molecules29174251/s1, Figure S1. The overall average mass spectra of the three leaf types of PRs by MALDITOF-MSI. (a) PT, (b) BT, and (c) WT. Figure S2. Detailed features of different samples in the Venn diagram.

## Abbreviations

PR: Pinelliae Rhizoma; PT, peach-leaf type; BT, bamboo-leaf type; WT, willow-leaf type; HPLC, high-performance liquid chromatography; LC-MS, Liquid Chromatography-Mass Spectrometry; MALDI, Matrix-Assisted Laser Desorption/Ionization; DESI, Desorption Electrospray Ionization; SIMS, Secondary Ion Mass Spectroscopy; MSI, mass spectrometry imaging; MALDI-TOF MS, Matrix-Assisted Laser Desorption/Ionization Time-of-Flight Mass Spectrometry; PCA, Principal Component Analysis; OPLS-DA, Orthogonal Projections to Latent Structures Discriminant Analysis; VIP, Variable Importance of Projection; 2-MBT, 2-mercaptobenzothiazole; MO-B, Methylophiopogonanone B; *P. ternata*, *Pinellia ternata*; *m*/*z*, mass-to-charge ratio; OCT, Optimum Cutting Temperature; TFA, trifluoroacetic acid; ITO, indium tin oxide.
