# Supplementary material for: Spatial Metabolomic Profiling of Pinelliae Rhizoma from Different Leaf Types Using Matrix-Assisted Laser Desorption/Ionization Mass Spectrometry Imaging
Source: Molecules. 2024 Sep 7;29(17):4251. doi: 10.3390/molecules29174251 (PMC11397683; doi:10.3390/molecules29174251)
Supplement: Supplementary file 1 [file molecules-29-04251-s001.zip › molecules-3144016-supplementary.pdf]

Supplementary Materials

Spatial Metabolomic Profiling of Pinelliae Rhizoma from Different Leaf Types Using Matrix-Assisted Laser Desorption/Ionization Mass Spectrometry Imaging

Jiemin Wang [1,2,3], Xiaowei Han [1,2,3], Yuguang Zheng [1,2,3], Yunsheng Zhao [1,2,3], Wenshuai Wang [1], Donglai Ma [1,2,3,*] and Huigai Sun [1,2,3,*]

[1] College of Pharmacy, Hebei University of Chinese Medicine, Shijiazhuang 050200, China; wjm15030331834@163.com (J.W.); hanxiaowei2015@126.com (X.H.); zyg314@163.com (Y.Z.); zwhjzs@126.com (Y.Z.); wangwenshuai2003@126.com (W.W.)

[2] Traditional Chinese Medicine Processing Technology Innovation Center of Hebei Province, Shijiazhuang 050200, China

[3] Key Laboratory for Quality Ensurance and Innovative TCMs of Dao-di Herbs, Hebei Provincial Administration of Traditional Chinese Medicine, Shijiazhuang 050200, China

* Correspondence: madonglai@hebcm.edu.cn (D.M.); sunhuigai@hebcm.edu.cn (H.S.)

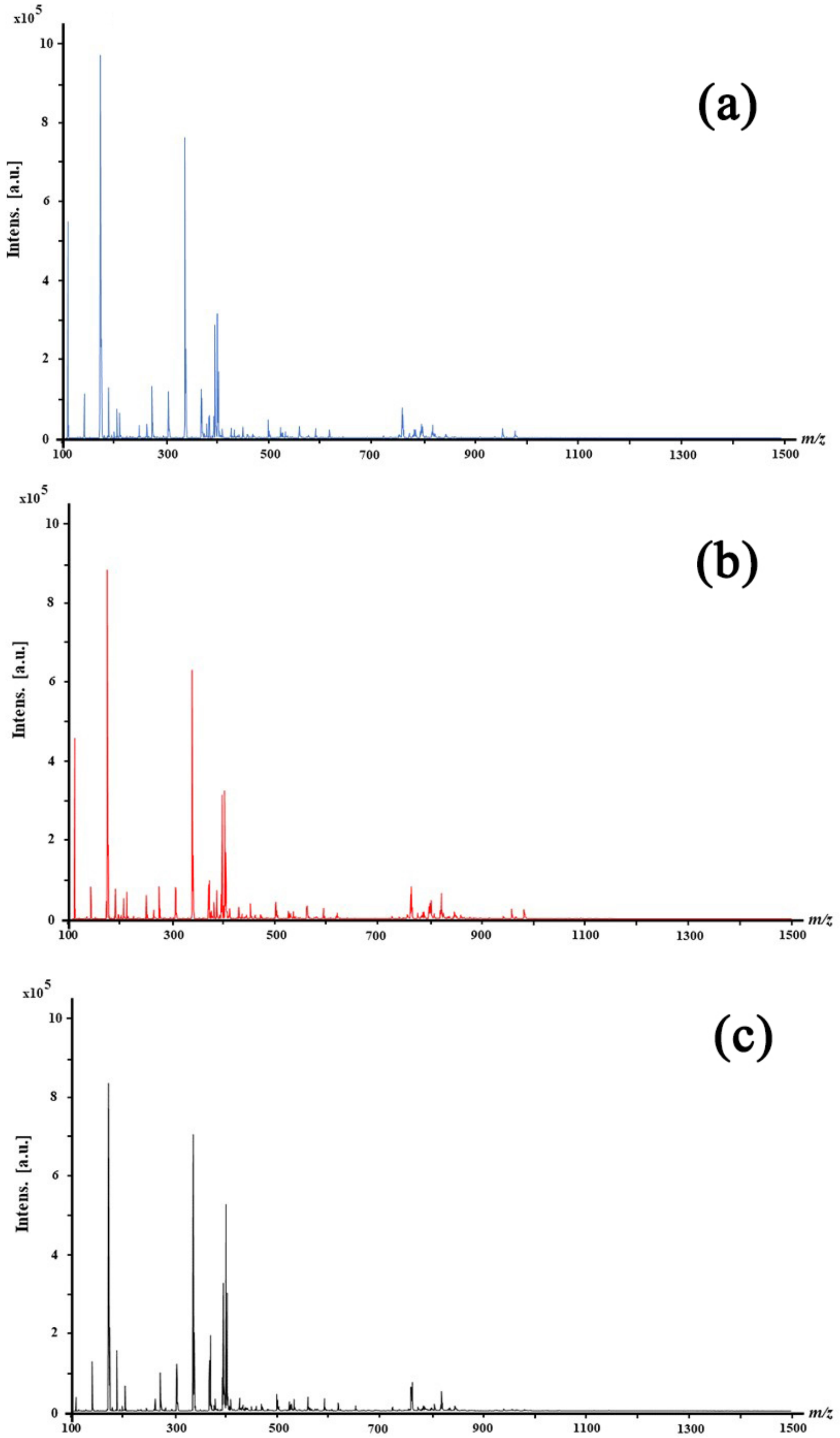

Figure S1. The overall average mass spectra of the three leaf types of PRs by MALDI-TOF-MSI. (a) PT, (b) BT, and (c) WT.

Names	total	elements
BT PT WT	275	276 847.5 206 858.5 455.2 801.5 305.2 446.2 532 512 458 980.5 454 572.1 964.5 482.4 603.1 325.3 347 557.1 438.3 269.1 761.6 291 199 521.4 404.1 337 802.5 805.5 784.6 198 387.2 772.4 480.3 406.1 834.5 794.5 781.5 820.5 202 563.9 589.1 368.2 558.1 124 862.5 577.5 258.1 502 524.4 754 722.1 559.1 576.5 863.6 788.6 335 270.1 262.3 783.6 229.9 756.5 621 796.5 272.2 184.1 498.4 817.5 334 403.1 981.5 844.5 773.4 201 423 575.5 843.5 497.4 170.1 587.1 467 527.4 792.6 434.3 422 261.1 558.3 302 800.5 561.9 861.6 195.1 370 762.6 439.2 137.045 196.1 402 104.1 778.6 787.6 293.1 520.4 376.1 377.1 799.5 187.1 231.9 365.9 983.5 175.1 271.1 373 690.1 785.6 965.5 408.1 573.1 966.5 539.1 274.2 399 848.5 757.5 541.1 301 436 430.4 940.6 775.5 138.1 456 394.1 169 736.5 171 398 184.8 372.1 200 110.1 125 808.5 190 279.1 400 828.5 378.2 845.5 804.5 468 226.026 259.1 744.5 525.4 397 379.1 496.4 381.1 225.1 599.5 424.1 407.1 393.1 304.1 821.5 724 842.5 147.1 523.4 212.1 280.3 435 401 427 822.5 955.5 856.4 333 392.1 296.1 806.5 209.1 590 531 982.5 172 819.5 114.1 391.1 617.2 105.1 425.1 428.9 831.5 979.5 816.5 774.5 260.1 779.6 478.2 760.6 128.1 426 369 522.4 956.5 431.4 498 759.6 616.2 798.5 723.1 336 457 500 939.6 182 136.1 957.5 1144.6 518.4 191.1 797.5 833.5 1145.6 367.1 167 818.5 156.1 167.5 592 257.1 571.1 396 758.6 499 530 166.1 555.1 365 186.1 470 469 374.1 273.3 375 941.5 963.5 786.6 285.1 437.4 466 244.1 429.4 185.4 501 830.5 405.1 846.5 390.1 618.3 782.6 526.4 746.5 832.5 780.5 303.2 185 332.1 227.1 591 298.1 242
BT WT	7	560 790.5 650.5 860.4 144.1 835.4 850.4
PT WT	10	428.4 967.6 565.2 165.1 734.5 124.7 925.5 151.1 275.3 169.5
BT PT	83	447.9 421.1 823.5 106.1 208 534.3 383.1 864.6 912.5 494.3 978.5 229.2 245.9 874.5 203.1 985.5 754.5 958.5 613.9 389.1 388 355 440.2 535.3 448.9 488 752 446.3 913.5 873.5 1146.4 330.1 984.5 306.3 751.4 338 245.077 560.4 977.5 448.3 755 189.1 316.1 824.5 112.985 911.5 753 875.5 461.2 795.5 857.4 397.4 371 959.5 753.5 1089.5 493.1 292.2 756 444.2 636 559.3 263.3 737.5 749.5 825.5 764.5 882.4 219.1 284.1 543.2 515.1 961.5 207 561.4 826.5 290 1090.5 449.4 104.7 814.5 382.1 752.5
WT	23	170.6 331 112.1 651.5 176.1 690.4 120.065 364 551.3 135.02 243.1 237 129.1 646.3 177 674.4 109 224 256 409.1 215.1 412.2 688.4
BT	41	540 561.3 872.3 1067.5 319.2 1107.4 1106.4 896.3 880.3 222 247.9 357.3 1068.5 532.9 1079.5 300.2 870.3 288.2 859.4 114.9 895.3 750.4 556.4 849.4 886.4 894.3 883.3 884.3 868.3 840.4 286.2 615.9 854.3 432.3 738.5 1024.6 192 1105.4 614.9 394.9 162.9
PT	16	810.6 318.3 871.6 812.6 254.2 953.5 241.2 815.5 604.1 643.7 146.2 239.1 1147.2 642.7 942.5 1009.4

Figure S2. Detailed features of different samples in the Venn diagram.

Supplementary Materials-experimental section

Instruments and materials

Agilent 1260 high performance liquid chromatography instrument. Uracil (HPLC≥98%, Lot. No. 612A027), cytidine (HPLC≥98%, Lot. No. 1114A027), uridine (HPLC≥98%, Lot. No. 610B022), 6-Hydroxypurine (HPLC≥98%, Lot. No. 606C021), inosine (HPLC≥98%, Lot. No. 729B025), guanosine (HPLC≥98%, Lot. No. 1028B023), thymidine (HPLC≥98%, Lot. No. A315A024) and adenine (HPLC≥98%, Lot. No. 912A029) 〔Beijing Solarbio Science & Technology Co., Ltd. Beijing, China (https://solarbio.com/). Methanol and acetonitrile are chromatographically pure, other reagents are analytically pure, and water is ultrapure water.

Liquid chromatography condition

Chromatographic column: Agilent ZORBAX SB-Aq (4.6 mm x 250mm, 5μm); Moving phase：C_2H_3N (A)-H_2O (B) gradient elutio (0~7min, 0%A; 7~13min, 0%~12%A; 13~18min, 12%A~25%A, 18~35min, 25%~60%A; 35~40min, 60%~0%A); Flow velocity: 1.00 mL·min^{-1}; Sample size: 20μl; Column temperature: 30°C; Check wavelength: 265nm。

Solution preparation

Preparation of reference solution

Accurately weigh uracil, cytidine, uridine, 6-Hydroxypurine, inosine, guanosine, thymidine and adenine as 1.31, 1.00, 1.19, 1.07, 1.04, 1.56, 1.13 and 1.11mg, then put them into eight 5mL volumetric bottles and dissolve them with water

Preparation of test product solution

Put 1. 0000g of powder of each sample into 100 mL triangle bottle, add 50 mL ultra-pure water, and weigh. After ultrasonic extraction (500 W, 40 kHz) for 45 min, the weight was made up with ultrapure water. After centrifugation at 12 000 r/min for 10 min, the supernatant was taken into a triangular flask; the residue was transferred into the triangular flask, 50 mL of ultrapure water was added and weighed. After ultrasonic extraction (500 W, 40 kHz) for 45 min, the supernatant was removed by centrifugation with ultrapure water to make up the weight. The residue was extracted once more and the supernatant of the three extracts was combined. 50 °C water bath was used to concentrate the supernatant to a final volume of 10 mL and set aside. Pass through 0. 22 μm aqueous microporous membrane and use.
